# Adult paroxysmal cold hemoglobinuria following mRNA COVID‐19 vaccination

**DOI:** 10.1002/jha2.508

**Published:** 2022-06-15

**Authors:** Kyohei Misawa, Hajime Yasuda, Daisuke Koyama, Tadaaki Inano, Akemi Inoguchi, Chinami Shirasu, Hina Takano, Noriaki Iwao, Miki Ando, Michiaki Koike

**Affiliations:** ^1^ Department of Hematology Juntendo University Shizuoka Hospital Izunokuni Shizuoka Japan; ^2^ Department of Hematology Juntendo University Graduate School of Medicine Bunkyo‐ku Tokyo Japan; ^3^ Department of Hematology Fukushima Medical University Fukushima Fukushima Japan; ^4^ Department of Clinical Laboratory Medicine Juntndo University Shizuoka Hospital Izunokuni Shizuoka Japan

**Keywords:** autoimmune disease, COVID‐19 vaccination, paroxysmal cold hemoglobinuria

## Abstract

Paroxysmal cold hemoglobinuria (PCH) is an extremely rare subtype of autoimmune hemolytic anemia (AIHA) in adults. PCH is caused by the biphasic Donath–Landsteiner (DL) antibody which fixes complement to red blood cells at low temperatures and dissociates at warmer temperatures, leading to complement‐mediated intravascular hemolysis. Autoimmune hematological disorders including AIHA and immune thrombocytopenia have been reported to develop following the mRNA COVID‐19 vaccination. However, PCH developing subsequent to mRNA vaccination has never been reported. We report a 59‐year‐old male who developed PCH approximately a month after his second mRNA COVID‐19 vaccination.

## 1 INTRODUCTION

Autoimmune hemolytic anemia (AIHA) is a rare disorder and can be classified into five subtypes [1]. Three of the AIHA subtypes are cold‐types of AIHA, which are cold agglutinin disease (CAD), secondary cold agglutinin syndrome (CAS), and paroxysmal cold hemoglobinuria (PCH).

PCH is caused by an autoantibody that fixes complement to red blood cells (RBCs) at low temperatures and dissociates at warmer temperatures, leading to complement‐mediated intravascular hemolysis. This biphasic antibody is known as the Donath–Landsteiner (DL) antibody and its presence is essential for the diagnosis of PCH. There are many pediatric cases of PCH, while adult cases are extremely rare [2]. Most adult PCH cases were known to be caused by syphilis in the early 1900s. The numbers of PCH patients have drastically fallen with the decrease of untreated syphilis patients. Reports of PCH are scarce in recent years, and the causes of PCH were reported to be idiopathic or due to hematological malignancies including non‐Hodgkin lymphoma (NHL), chronic lymphocytic leukemia, myelofibrosis, and myelodysplastic syndrome (MDS) [3, 4, 5, 6].

Autoimmune hematological disorders including AIHA and immune thrombocytopenia have been reported in relation to COVID‐19 infections and vaccinations [7, 8, 9, 10, 11]. However, PCH has not been previously reported in association with the mRNA COVID‐19 vaccine.

## 2 CASE REPORT

**FIGURE 1:**
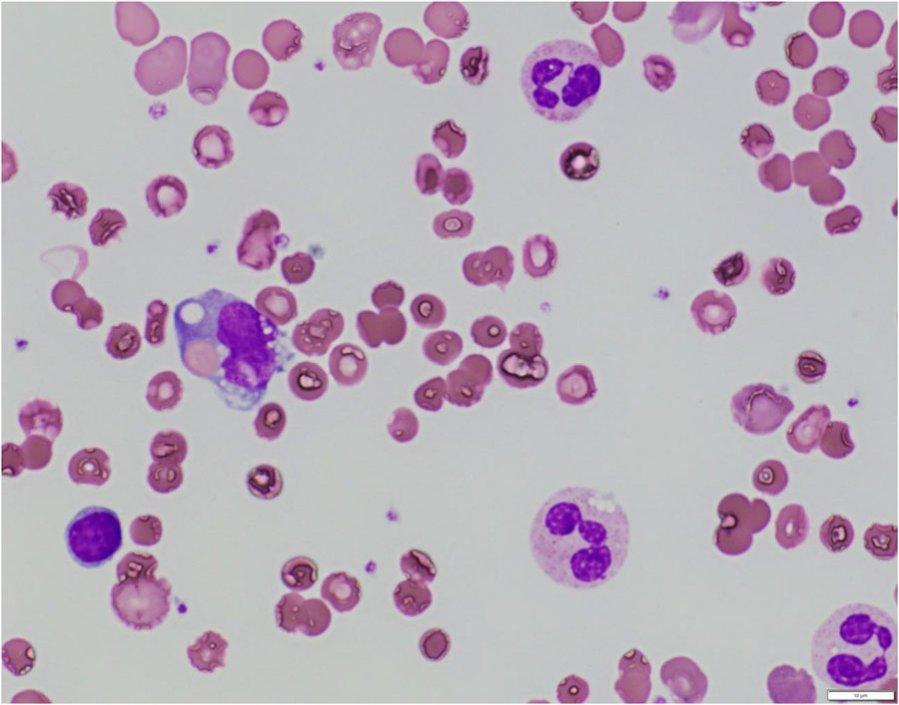
Neutrophil erythrophagocytosis and red cell agglutination in peripheral blood smear

**FIGURE 2:**
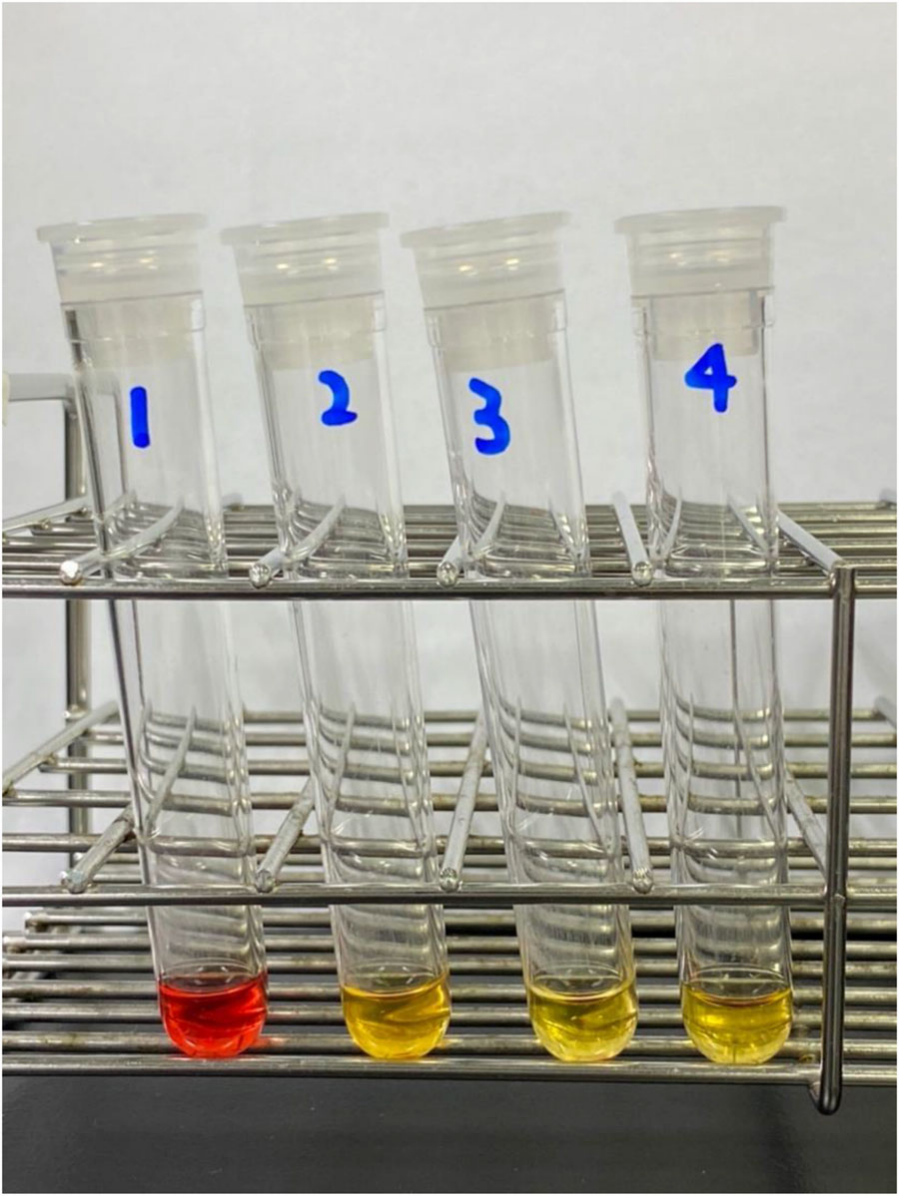
Results of the Donath–Landsteiner test. Patient's serum was obtained from the peripheral blood after being centrifuged at 37°C. P‐antigen‐positive group O RBCs were added to all tubes and each tube consists of the following; Tube 1: patient's serum + donor fresh serum incubated at 0°C for 30 min and subsequently at 37°C for 30 min. Tube 2: patient's serum + donor fresh serum incubated at 37°C for 30 min. Tube 3: donor fresh serum incubated at 0°C for 30 min and subsequently at 37°C for 30 min. Tube 4: donor fresh serum incubated at 37°C for 30 min

**FIGURE 3:**
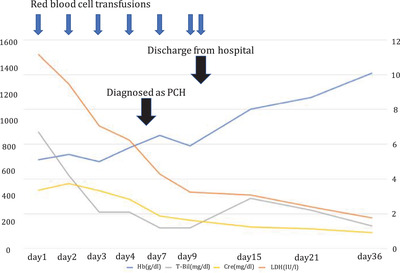
Clinical course of the patient. Days are counted as the days from first visit to our hospital. Abbreviations: Cre; creatine, Hb, hemoglobin; LDH, lactate dehydrogenase; T‐Bil, total bilirubin. Only LDH values are dealing with the left axis

A 59‐year‐old Japanese male was admitted to our institution in early December, 2021 due to a two‐week history of high fever, fatigue, and shortness of breath. Onset of these symptoms appeared roughly four weeks after the second dose of BNT162b2 mRNA COVID‐19 vaccination (Pfizer‐Biotech). PCR tests for COVID‐19 were negative, and peripheral blood tests showed severe anemia with hemoglobin of 5.1 g/dl, high total bilirubin levels of 6.7 mg/dl, and high lactate dehydrogenase levels of 1489 IU/L. Hemoglobinuria was present. Acute renal failure was also observed and creatinine (Cr) was 3.37 mg/dl. Peripheral blood smear showed agglutination of RBCs and active neutrophil erythrophagocytosis (Figure 1). Direct antiglobulin test (DAT) was positive for complement C3b/C3d, and negative for IgG (Ortho BioVue^Ⓡ^ System, DAT/IDAT Cassette). The direct DL test was negative, but the indirect DL test (done by mixing patient and donor serum and adding P‐antigen‐positive type O RBCs) was positive (Figure 2). Tests for Syphilis and cold agglutinin titers were negative, and bone marrow analysis showed no remarkable abnormalities. Whole body computed tomography (CT) scans were also unremarkable and showed no signs of malignant lymphoma. Blood and urine cultures were negative, and serological tests denied active infections of Epstein–Barr virus, cytomegalovirus, HIV, HTLV‐1, and Mycoplasma pneumoniae. Thus, COVID‐19 vaccination‐related PCH was suspected. As for treatment, RBC transfusions and hydration were carried out, and the patient was kept warm. Anemia, LDH levels, and renal failure gradually improved, and the patient was discharged 10 days from admission. On his first outpatient visit, his general condition was well, and laboratory data showed improved hemoglobin levels and normalization of renal function. Two months after first outpatient visit, hemoglobin levels normalized but the indirect DL test remained positive (Figure 3).

## 3 DISCUSSION

We encountered a case of adult onset PCH with no preceding infections or LPDs. PCH is a rare immunologic disorder often associated with infections and LPDs. Although syphilis and viral infections are known to trigger production of DL antibodies, the underlying mechanisms have not been fully elucidated. In general, the DL antibody targets the P‐antigen on the RBC surface, but certain rare cases have reported targeting of the i‐antigen [6]. Recently, certain autoimmune hematological disorders including warm‐type AIHA, CAS, and PNH have been reported to be triggered by COVID‐19 vaccinations [7, 8, 9, 10, 11]. Thus in theory, PCH is also a likely disorder to be triggered by COVID‐19 vaccinations due to its autoimmune nature. Although there were two case reports of PCH onset following measles and seven‐valent pneumococcal conjugate vaccination [12, 13], to the best of our knowledge, this is the first to report development of PCH subsequent to the mRNA COVID‐19 vaccination. Symptoms of hemolysis were reported to develop a few days to a month after mRNA COVID‐19 vaccinations in the aforementioned cases of AIHA, CAS, and PNH. Our case developed symptoms of hemolysis approximately 5 weeks after the second COVID‐19 vaccination, which is relatively late as for timing of onset compared to most reported cases of autoimmune hematological disorders triggered by vaccination. However, the patient received his second COVID‐19 vaccination in middle October, and symptom onset was in late November. We speculate that the patient may have developed DL antibodies earlier, but did not experience hemolysis until late November because this is the time of the year when the temperature usually starts to fall in Shizuoka prefecture, which is located in the middle of main island Japan.

Due to the decline in number of patients with syphilis, adult cases of PCH have become an extremely rare disorder, and thus PCH can be overlooked by current day practitioners. Moreover, other than hemolysis‐associated manifestations such as hemoglobinuria and jaundice, the symptoms of PCH are diverse and none are disease specific, including myalgias, abdominal pain, back pain, anorexia, headaches, rigors, nausea, and fever. Furthermore, although confirmation of the DL antibody is essential for a diagnosis of PCH, the DL test requires careful prewarmed sample collection and a highly skilled technician to acquire accurate results. The direct DL test is prone to false‐negative results because the complement coated cells show resistance to lysis. Therefore, when PCH is strongly suspected, the indirect DL test should be additionally performed even when the direct DL test is negative. Another important diagnostic clue is peripheral blood erythrophagocytosis, which is highly specific to PCH. In the presented case, DAT was positive for complement C3b/C3d and negative for IgG, the indirect DL test was positive, erythrophagocytosis was observed in the peripheral blood smear, and thus a firm diagnosis of PCH was rendered.

Management of PCH is fundamentally supportive, constituted by methods such as hydration, RBC transfusions, and avoiding cold exposure. Use of infusion warmers should be considered when blood transfusions and fluid infusions are to be carried out. Although immune‐suppressive therapies including corticosteroid, rituximab, intravenous immunoglobulin (IVIG), and azathioprine have been used for severe or chronic hemolysis due to PCH, the true efficacy of these agents are unclear. Eculizumab, a humanized anti‐C5 monoclonal antibody which blocks the complement pathway at the C5 stage, has been looked upon as a promising treatment method for PCH [14]. In the presented case, hemolysis promptly improved with warming alone and no recurrence has been seen, and thus additional therapies were not necessary.

In conclusion, we report the first case of PCH developing subsequent to the mRNA COVID‐19 vaccination. Several autoimmune hematologic disorders have been reported following the mRNA COVID‐19 vaccination, and PCH may be an underrecognized adverse event. Although we could only provide circumstantial evidence and no solid proof that mRNA COVID‐19 vaccination contributed to the development of PCH, adult PCH in the current era is extremely rare and the possibility of PCH developing coincidentally following vaccination can be considered highly unlikely. Accumulation of cases and further etiological studies are necessary for understanding the exact relationship between mRNA COVID‐19 vaccination and PCH.

## FUNDING

No funding was provided for this study.

## CONFLICT OF INTEREST

The authors declare that there is no conflict of interest that could be perceived as prejudicing the impartiality of the research reported.

## AUTHORS CONTRIBUTION

Kyohei Misawa: conceptualization, data collection and interpretation, writing the original draft; Hajime Yasuda: investigation, writing the original draft; Daisuke Koyama: supervision, data collection and interpretation, revising the manuscript; Tadaaki Inano: conceptualization, data collection and interpretation; Akemi Inoguchi: data interpretation and providing of technical support; Chinami Shirasu: data interpretation and providing of technical support; Hina Takano: conceptualization, data collection and interpretation, Noriaki Iwao: data collection and interpretation, supervision; Miki Ando: supervision, revising the manuscript; Michiaki Koike: supervision, revising the manuscript.

## ETHICS STATEMENT

This study was approved by the ethics committee of Juntendo Shizuoka Hospital. Written informed consent for this study was obtained from the patient.

## Data Availability

Not applicable as no datasets were generated for this study.
